# The Association of Health-Promoting Lifestyle With Quality of Life Among the Iranian Elderly

**DOI:** 10.5812/ircmj.18404

**Published:** 2014-09-05

**Authors:** Tayebeh Rakhshani, Davood Shojaiezadeh, Kamran Bagheri Lankarani, Fatemeh Rakhshani, Mohammad Hossain Kaveh, Najaf Zare

**Affiliations:** 1 Department of Health Education and Promotion, Tehran University of Medical Sciences, Tehran, IR Iran; 2 Health Research Center, Shiraz University of Medical Sciences, Shiraz, IR Iran; 3 Safety Promotion and Injury Prevention Research center, Shahid Beheshti University of Medical Sciences, Tehran, IR Iran; 4 Department of Health Education and Promotion, Shiraz University of Medical Sciences, Shiraz, IR Iran; 5 Department of Biostatistics, Shiraz University of Medical Sciences, Shiraz, IR Iran

**Keywords:** Quality of Life, Health-Promoting Lifestyle, the Elderly

## Abstract

**Background:**

As individuals live a longer life, health-promoting lifestyle (HPL) becomes even more essential, particularly with regard to maintaining functional independence and improving quality of life (QoL).

**Objectives:**

This study aimed to determine the association between QoL and HPL in the Iranian elderly living in Shiraz.

**Materials and Methods:**

This analytical cross-sectional study was conducted in 2013 at retirement centers of Shiraz City, Iran. The sample included 500 elderly who aged > 60 years. Proportional stratified random sampling was used to select the elderly from retirement centers. QoL was assessed by the Farsi version of Short Form Health Survey questionnaire (SF-36) and HPL was measured by health-promoting lifestyle profile (HPLP II). Data were analyzed using independent-samples t test, one-way ANOVA, Pearson’s correlation, and stepwise multiple regression using SPSS 21.

**Results:**

There were significant differences in QoL in terms of sex, age, education, and marital status. There were significant differences in HPL in terms of gender, age and education (P < 0.05) There was a statistically significant association between QoL and HPL in the elderly (r = 0.42, P < 0.05). Based on HPLP II constructs, the significant predicting factors of QoL in the elderly consisted of spiritual growth, stress management, and physical activity (P < 0.05).

**Conclusions:**

Health providers should improve the QoL in the elderly by facilitating HPL through health-promoting interventions, which will maintain and increase physical activity, stress management, and spiritual growth.

## 1. Background

The world’s population is aging (1). The improvement in the living conditions has led to the phenomena of aging in different societies (2, 3). Like other countries, Iran has experienced a shift in the population structure towards aging so that according to statistics in 2011, 8.19% of Iran’s population were older than 60 years (4).

As aging is considered an important social issue worldwide, the biggest challenge is improving the quality of life (QoL) in the elderly (5, 6). QoL is a multidimensional concept that includes the individual’s physical, psychological, and social performances (7). The increase of human’s life span emphasis the importance of the health-promoting behavior in maintaining and improving QoL (8-10). The World Health Organization has placed an emphasis on the importance of health-promoting behavior as a key strategy for maintaining a good QoL (11). Pender et al. classified the health-promoting lifestyle (HPL) into six subcategories of nutrition, physical activities, stress management, health responsibility, interpersonal relations, and spiritual growth (12).

In Iran, researches have been conducted on different age groups, except the elderly, regarding the association between HPL and QoL (13, 14). In developed countries, the association of one or two subscales of HPL with QoL in the elderly have been investigated, which have mainly dealt with the aspect of nutrition and physical activity and the results have indicated that nutrition and physical activities are effective factors in QoL of the elderly (15-18). 

A study in China indicated that interpersonal relations, spiritual growth, and physical activities were better predictors for QoL in older than 50 years of age retired people (19, 20). Despite some similarities between the elderly in Iran and the elderly in developed countries, the cultural, religious, and environmental issues should not be neglected (21). In Iran, the results of a study by Aghanuri et al. has indicated that the quality of nutrition does not have a significant association with the QoL in the elderly (22). On the contrary, researches in developed countries show a significant association between nutrition and QoL in the elderly (23-25). Knowledge about the factors that influence QoL is important as population aging becomes a worldwide reality (8). There is less evidence about the amount of influence of HPL on the health-related QoL (HRQoL) in the Iranian elderly and it should be considered that any designed and executed strategy for health promotion needs evidence-based science, because if there is no awareness and recognition of the status of the society’s health factors, health programs may just impose high costs. Hence, the results of this study can help health specialists, managers, and policymakers to design and execute health-promoting strategies based on the evidences, and prepare a basis for further researches in the field of aging.

## 2. Objectives

This study aimed to investigate the association between HPL and its subscales (nutrition, physical activities, stress management, health responsibility, interpersonal relations, and spiritual growth) with QoL in the Iranian elderly. Moreover, this study familiarized us with the status of the elderly's QoL and HPL.

## 3. Materials and Methods

### 3.1. Design

This research was an analytical cross-sectional study to determine the association between QoL and HPL among the elderly in Shiraz, Iran, in 2013. 

### 3.2. Participants and Setting

The research population included the retired elderly or their spouse that were members of the state retirement centers. The inclusion criteria were retired males or females with ≥ 60 years of age, who were able to speak, had no hearing impairment or any serious health problems, and were willing to participate in the study.

### 3.3. Sampling and Data Collection

A total of 45671 retired elderly were registered in the state retirement centers. The sample size was estimated at 500 by using Morgan formula. The sampling method was based on a proportionate stratified random sampling approach. We considered those retirement centers with the elderly members > 200 who had agreed to participate in the study as one stratum.

Considering the sample size, proportionate allocation sampling was used to identify a sampling fraction for each center. The participants were selected randomly from existed elderly members’ list in each center. Participate in the study were interviewed face to face by trained interviewers with a standard questionnaires at the appropriate time and place so there was no missing data in this study.

### 3.4. Measurement Tools

The Farsi version of Short Form Health Survey questionnaire (SF-36) was used to collect data on QoL. The total score of SF-36 ranges from zero to 100 and higher scores indicate a better condition. The reliability and validity of this scale has been approved for the elderly in some surveys (26, 27). The obtained Cronbach’s alpha was 0.93 for QoL in this study. The Health-promoting Lifestyle Profile (HPLP II) is a 52-item rating scale and consists of six subscales to measure major aspects of a health promoting lifestyle including health responsibility, physical activity, nutrition, interpersonal relations, spiritual growth, and stress management. The mean can be obtained on the total scale as a measurement of HPL and a mean can be derived for each subscale. A mean score higher than the average score was considered as the better response by the authors. The reliability and validity of this scale has been approved for the elderly in some studies (27, 28). In this study, the specialist group assessed the content validity of the Farsi version of HPLP II scale and found it to be culturally relevant with the obtained alpha coefficient of 0.93.

### 3.5. Data Analysis

Frequencies, percentage, mean, and standard deviation were used to describe demographic characteristics, QoL, and HPL. Independent-samples t-test and ANOVA with post hoc test were used to determine the differences in QoL and HPL. Pearson’s correlation coefficient was used to identify the degree of correlation between QoL with HPL and its subscales. In addition, stepwise multiple regressions were used to determine the predicting factors of QoL with respect to HPLP II subscales. A P value < 0.05 was considered as statistically significant. As part of the regression analysis, the assumption of normality, linearity, homoscedasticity, and independence of residuals were tested using residual scatter plots, correlation matrix, Durbin-Watson, tolerances, and variance inflation factor, respectively.

### 3.6. Ethical Considerations

The Ethic Committee of Tehran University of Medical Sciences approved the study protocol (date, 2013-05-19; ID, 240/32251). All participants gave their oral consents for interview. We kept the information of the participants confidential.

## 4. Results

Overall, 53.6% of the participants were male and 46.4% female. The highest frequencies belonged to the age group of 60 to 75 years (76.4%), the elementary level (39.4%), and the married (73%) in age groups, education level, and marital status, respectively (Table 1). Table 2 shows the possible ranges, means, and standard deviations of HPL, its subscales, and QOL. The mean QoL score of the participants was 50.8 ± 17.01 and the mean score of HPL was 122.78 ± 23.34. In addition, interpersonal relationship and spiritual growth had the highest score and physical activity had the lowest score in the HPLP II subscales of the elderly (Table 2). According to independent-samples t-test and ANOVA, there were significant differences in QoL and HPLP II scores in terms of sex, age, and education whereas marital status had significant differences on QoL (P < 0.05) not on HPLP II (P > 0.05). The means scores of QoL and HPLP II of male and age group < 75 years old were significantly higher than the means scores of QoL and HPLP II of female and age group ≥ 75 years old (P < 0.05). Post hoc testing indicated that the means scores of QoL and HPLP II of those with lower education (Elementary education) were significantly lower than that of the other education levels in the elderly (P < 0.05). The mean score of QoL of married individuals was significantly higher than that of widowed/divorced ones (P < 0.05) (Table 3).

Pearson correlation coefficient was used to describe the magnitude and direction of the association between QoL and HPLP II subscales. There was a statistically significant correlation between QoL and HPLP II and its subscales in the elderly (P < 0.001) at the 0.01 level. Results are summarized in (Table 4).

Multiple linear regression analysis with stepwise method showed that three subscales of HPLP II including spiritual growth, stress management and physical activity were significant predicting factors of QoL in the elderly; they explained 31.4% of the variance of QoL while the other subscales of HPLP II explained 1% of it (adjusted R^2^ = 0.314, R^2^ change = 0.01). The level of statistical significance was at P < 0.05 (Table 5).

**Table 1 .** The Socio-Demographics Characteristics of the Elderly participants (n = 500) ^a^

Characteristics	Results
**Sex**	
Female	232 (46.4)
Male	268 (53.6)
**Age**	
< 75	382 (76.4)
≥ 75	118 (23.6)
**Education**	
Elementary	197 (39.4)
High School	190 (38)
Diploma	81 (16.2)
University Education	32 (6.4)
**Marital Status**	
Married	365 (73)
Single	17 (3.4)
Windowed/Divorced	118 (23.6)

^a^ Data are presented as No. (%).

**Table 2.** The Range of Dimension of Health Promoting Lifestyle Profile in Elderly (n = 500) ^a,b^

Variables	Results	Median (Range)
**Total HPLP II Score**	122.78 ± 23.34	125.5 (52-208)
**Physical Activity**	14.66 ± 4.71	20 (8-32)
**Stress Management**	19.27 ± 4.4	20 (8-32)
**Health Responsibility**	19.6 ± 5.22	22.5 (9-36)
**Nutrition**	22.41 ± 4.93	22.5 (9-36)
**Spiritual Growth**	23.12 ± 5.6	22.5 (9-36)
**Interpersonal Relationship**	23.69 ± 4.62	22.5 (9-36)
**Total SF-36 Score**	50.88 ± 17.01	50 (0-100)

^a^ Data are presented as mean ± SD.

^b^ Abbreviations: HPLP II; health-promoting lifestyle profile, SF-36; short form health survey questionnaire.

**Table 3.** The Differences of Scores in Quality of Life and Health-Promoting Lifestyle Based on Demographic Variables in the Elderly (n = 500) ^a,b^

	Quality of Life (SF-36)			Health Promoting Lifestyle (PHLP II)		
	Mean ± SD	T a, F b Statistics	P Value	Mean ± SD	T a, F b Statistics	P Value
**Sex**		t = -6.66	0.00		t = -3.08	0.002
Male	55.4 ± 17.2			125.75 ± 23.96		
Female	45.7 ± 15.3			119.34 ± 22.16		
**Age**		t = 4.59	0.00		t = 2.37	0.01
< 75	52.79 ± 16.57			124.15 ± 22.54		
> 75	44.71 ± 17.02			118.34 ± 25.37		
**Education **		F = 17.34	0.00		F = 16.6	0.00
Elementary	45.5 ± 16.12			114.22 ± 20.03		
High School	52.65 ± 15.75			128.64 ± 21.42		
Diploma	56.63 ± 16.61			126.01 ± 26.75		
University Education	61.71 ± 18.66			134.5 ± 28.08		
**Marital Status**		F = 18.06	0.00		F = 2.33	0.09
Married	53.5 ± 16.63			124.14 ± 23.3		
Single	49.01 ± 24.51			118.64 ± 32.15		
Windowed/Divorced	43.06 ± 14.42			119.15 ± 21.71		
**Total**	50.88 ± 17.01			122.78 ± 23.34		

^a^ Acronyms: HPLP II, health-promoting lifestyle profile; and SF-36, short form health survey questionnaire.

^b^ Independent-samples t-test (P < 0.05) and One-way ANOVA (P < 0.05) were used.

**Table 4 .** The Correlation Between Quality of Life and the Dimensions of Health Promotion Lifestyle Profile in the Elderly (n = 500) ^a^

Variable	QoL (SF-36 Score)	
	R ^b^	P Value
**Total HPLP II Score**	0.47 ^c^	0.00
**Spiritual Growth**	0.52 ^c^	0.00
**Stress Management**	0.46 ^c^	0.00
**Physical Activity**	0.41 ^c^	0.00
**Interpersonal Relationship**	0.35 ^c^	0.00
**Health Responsibility**	0.27 ^c^	0.00
**Nutrition**	0.2 ^c^	0.00

^a^ Abbreviations: QoL; quality of life, SF-36; short form health survey questionnaire, HPLP II; health-promoting lifestyle profile.

^b^ Pearson correlation coefficient.

^c^ P < 0.01.

**Table 5.** Predicting Factors of Quality of Life Among the Elderly by Stepwise Multiple Regression Analysis (n = 500) ^a^

Variable	Unstandardized Coefficients		Standardized Coefficients	t	P Value
	B	Std. Error	Beta		
**Spiritual Growth**	1.17	0.16	0.38	7.05	0.00
**Stress Management**	0.65	0.22	0.17	2.87	0.00
**Physical Activity**	0.59	0.17	0.16	3.45	0.00

^a^ B0 = 13.73; R = 0.56; R^2^ = 0.32; Adjusted R^2^ = 0.314; R^2^ change = 0.01; (F = 58.19, P value = 0.00).

## 5. Discussion

In general, the obtained scores of SF-36 in this study showed moderate QOL in the elderly. These result were compatible with the QOL of the elderly in Tehran (29). The obtained scores of HPLP II were a little lower than the moderate scores; the HPL condition in older than 50 years old Chinese people was similar to that of our study population (20). We could find no data on HPL condition among the elderly in the other cities of Iran; therefore, it is better to conduct further studies on the HPL status in the Iranian elderly.

In this study, the participants obtained fairly higher scores for spiritual growth as well as interpersonal relationship and fairly lower scores for nutrition, stress management, and health responsibility. In addition, the lowest score belonged to physical activity, which is emphasizing and contemplating. One possible reason may be that elderly do not consider health-promoting behavior as being necessary for healthy aging while the recent evidence supports that healthy lifestyle is an effective strategy for sound aging (30). 

In the study on retired workers, the highest and lowest scores belonged to the interpersonal relations and health responsibility, respectively (20). A study in elderly women determined that the lowest and highest scores belonged to physical activities and nutrition, respectively (31). With regard to these results, the elderly do not have enough health-promoting behavior, especially in physical activity, and further studies are needed to explore the cause of this problem. Considering the demographic features of the subjects, the QoL score was lower in females, older ages, single elderly, and those with lower educational level. These results were compatible with the results of many other studies in Iran and other countries (8, 29, 31-38). Moreover, HPLP II score was higher in males, < 75 years of age, and those with higher educational level. These results were compatible with the results in the other studies (19, 31). Therefore, it is better to pay attention seriously to the health status of the elderly that are female, older, and unmarried with lower education. Moreover, this study showed that HPL and all of its subscales are significantly associated with QoL in the elderly, which was consistent with those reported in China (19). These are similar with the results of other researches on other age groups in Iran (13, 14). The subscales with the highest correlations with HRQoL were spiritual growth, stress management, physical activity, and interpersonal relations, consecutively. In addition, nutrition and health responsibility had the lowest correlations with HRQoL. These results are consistent with the results of the study by Zhang (19). The findings by Keller et al. in Canadian elderly indicated that nutrition is significantly associated with the QOL and suggested intervening studies for confirming these results (24). Considering different correlation degrees of HPL subscales with QOL, the stepwise multiple regression analysis showed that spiritual growth, stress management, and physical activity were stronger predictors of QoL. A longitudinal study in Florida introduced physical activity as an effective predictor of QOL in the elderly (39). Another study in Brazil concluded that physical activity was an effective factor for QoL (38). In Chinese elderly, health responsibility, spiritual growth, and physical activity were stronger predictors of QOL (20). Hence, physical activity is considered as a positive effective factor for QoL in elderly of different societies. A study in Spain is another proof to this claim that suggested education and training on physical activities for improving QoL in the elderly (40). Spiritual growth was one of the important factors for improving QoL and the score of spiritual growth was higher than average in this study. Hence, it can be concluded that the tendency of most Iranian elderly towards religious rites and Islamic ceremonies and spending most of their times in the mosques can be the reason for better scores of spiritual growth. Researches have shown that spiritual growth plays an important role on QoL in the elderly and is related to their health (41). Despite the advance of scientific literature, more studies are required about the influence of spiritual performance on health (42).

### 5.1. Final Conclusion

With regard to the low level of health-promoting behaviors, especially physical activities and stress management that were stronger predictors of QOL in this study, physical activities and stress management must be included in health interventions in order to improve QoL in the elderly. It is recommended that the health policymakers adopt more strategies for health promotion in the elderly women due to their low QoL as women have an important role in the development of the communities.

### 5.2. Limitation and Strong Points

This study was a cross-sectional study with small sample; therefore, results could not be generalized to the entire elderly population in Iran. Interventional or longitudinal studies are recommended to confirm these results. The strong point of the study was simultaneously determining the association of the six HPL subscales, i.e. health responsibility, physical activity, nutrition, interpersonal relations, spiritual growth, and stress management, with QoL in the elderly. 
